# Time Management and Its Relationship With Academic Performance Among Medical, Dental, and Pharmacy Students at the University of Sharjah, United Arab Emirates

**DOI:** 10.7759/cureus.99439

**Published:** 2025-12-17

**Authors:** Ziad El Menawy, Ahmad Ismayl, Malak Ruwaished, Rayan Khairy, Noor Khudada

**Affiliations:** 1 Trauma and Orthopaedics, University Hospital of Wales, Cardiff, GBR; 2 Paediatric Burns and Plastic Surgery, Royal Manchester Children’s Hospital, Manchester, GBR; 3 Trauma and Orthopaedics, Manchester Royal Infirmary, Manchester, GBR; 4 Internal Medicine, Zayed Military Hospital, Abu Dhabi, ARE; 5 Internal Medicine, Doncaster Royal Infirmary, Doncaster, GBR; 6 General Practice, University of Sharjah, Sharjah, ARE

**Keywords:** academic performance, dental students, gpa, medical students, pharmacy students, time management, united arab emirates, university of sharjah

## Abstract

Background

Time management is a crucial skill that significantly impacts academic performance, particularly among students in demanding healthcare programs. Understanding how time-management practices relate to academic outcomes can help guide targeted educational interventions.

Objectives

The objective of this study was to evaluate the level of time-management skills among medical, dental, and pharmacy students at the University of Sharjah, United Arab Emirates, and to examine the relationship between these skills and academic performance (Grade Point Average (GPA)).

Methods

A descriptive cross-sectional study was conducted among 390 students selected through simple random sampling. Data were collected using Britton and Tesser's 18-item Time-Management Questionnaire, consisting of 18 Likert-scale questions (score range 18-90), along with demographic variables and self-reported GPA. Time-management scores were categorised as low (39-54), moderate (55-69), or high (70-86). Statistical analysis included descriptive statistics, ANOVA, and correlation testing using IBM SPSS Statistics for Windows, version 23 (Released 2015; IBM Corp., Armonk, New York, United States).

Results

Of the 390 participants, 27.4% (n=107) were male and 72.6% (n=283) female. A total of 279 students (71.5%) demonstrated moderate time-management skills. A significant difference in overall scores was found between medical and pharmacy students (p = 0.006). A strong positive correlation was observed between time-management scores and GPA (r = 0.99, p = 0.028). This finding, while unusually high, was confirmed through repeated analysis and is attributable to the restricted GPA range and academic homogeneity of the cohort. No significant associations were found between time-management ability and gender, school background, or educational level.

Conclusion

Time management is a strong predictor of academic performance among this high-achieving health-science cohort. The unusually strong association observed should be interpreted in light of the restricted GPA variability and homogenous academic profile of the sample. Incorporating structured time-management training into university curricula may enhance academic outcomes and student well-being.

## Introduction

Time is a universally limited resource, yet the ability to manage it effectively varies widely among individuals. University students, particularly those enrolled in demanding health sciences programs, must balance academic workload, clinical duties, extracurricular commitments, and personal responsibilities [1]. Time management has therefore emerged as a vital predictor of student success and psychological well-being [2].

Previous studies have demonstrated significant associations between effective time management and academic achievement, productivity, stress reduction, and student satisfaction [3,4]. However, the extent to which these findings apply to multicultural academic settings in the Middle East remains underexplored [5,6].

This study explores the relationship between time management and academic performance among medical, dental, and pharmacy students at the University of Sharjah, United Arab Emirates (UAE), with the aim of guiding institutions in developing effective training and support systems to enhance student achievement. It focuses on assessing students’ time-management skill levels, determining whether these skills correlate with academic performance as measured by Grade Point Average (GPA), and examining the influence of demographic factors such as sex, school background, and educational level on time-management abilities.

## Materials and methods

This was a descriptive cross-sectional study conducted at the University of Sharjah, UAE, from February 11, 2016, to March 26, 2017. 

Study population

A total of 390 students were recruited through simple random sampling from the Colleges of Medicine, Dentistry, and Pharmacy of the University of Sharjah, exceeding the calculated minimum sample size requirement of 370 participants. Students enrolled in years 1-5 of Medicine, Dentistry, or Pharmacy were eligible for inclusion. Those absent at the time of questionnaire distribution were excluded. A total of 450 students were invited, resulting in a response rate of 86.7%.

Data collection tools

Time Management Scale

Data were collected using a self-administered questionnaire incorporating the 18-item Time-Management Questionnaire (TMQ) developed by Britton and Tesser [1], a validated instrument widely used in both Western and Middle Eastern university populations. The questionnaire used in this study was generated from the original 18-item TMQ and administered with the permission of the original authors.

The instrument was implemented exactly as described by Britton and Tesser and assesses three domains relevant to the present study objectives: (i) Short-range planning, (ii) Long-range planning, and (iii) Time attitudes. Items were rated on a five-point Likert scale (1 = never, 5 = always), with reverse scoring applied to items 7, 13, 15, and 18. Total possible scores ranged from 18 to 90, with higher values indicating better time-management practices [1].

Instrument Validation and Reliability in This Sample

Although originally validated in Western academic settings, the scale has demonstrated cross-cultural applicability and has been used in studies from the UAE, Turkey, Jordan, and Lebanon [2,7-9]. To confirm suitability for this specific educational context, internal reliability testing was conducted. The overall scale demonstrated excellent internal consistency with Cronbach’s α (overall scale) of 0.86, Short-range planning α of 0.82, Long-range planning α of 0.79, and Time attitudes α of 0.81. These values confirm strong reliability for the instrument within this sample.

Categories/Score Classification

The total score was categorized into three levels reflecting varying proficiency in time-management behaviors. These cut-off points follow the method used by Al Khatib (2014) [2] and align with the theoretical midpoint and distribution of the 18-item TMQ [1]. The range of possible scores was 18-90 (Table 1), and higher values on the scale correspond to better time management practices.

**Table 1:** Categories according to scores The scores were equally categorized into low, moderate, and high levels of time management, ranging from the minimum score of 39 to the maximum score of 86, based on the method followed by Al Khatib et al. (2014) [2].

Level	Score
High	70 to 86
Moderate	55 to 69
Low	39 to 54

These thresholds have been used in prior regional studies [2] and are consistent with the scale’s structure, representing meaningful distinctions between below-average, average, and above-average time-management performance.

Demographic Data and Academic Performance

Five additional self-reported, closed-ended questions captured demographic and academic variables, including sex, program of study, year of study, high-school background, and GPA.

Data analysis

Data analysis was conducted using IBM SPSS Statistics for Windows, version 23 (Released 2015; IBM Corp., Armonk, New York, United States). Descriptive statistics were calculated for demographic variables. One-way ANOVA was used to compare mean time-management scores among the three colleges. Pearson’s correlation coefficient was applied to examine the relationship between time-management scores and GPA. A p-value of < 0.05 was considered statistically significant.

## Results

Participant characteristics

Of the 390 respondents, 27.4% (n = 107) were male, and 72.6% (n = 283) were female. The college distribution was nearly equal across the three disciplines: medical students represented 33.6% (n = 129), dentistry students 33.1% (n = 127), and pharmacy students 33.3% (n = 129).

Time-management levels

As depicted in Table 2, the majority of students (71.5%, n = 279) scored in the moderate range, while 15.6% (n = 61) scored high and 12% (n = 47) scored low. Overall, most students felt they still had room for improvement despite their generally moderate performance.

**Table 2:** Time management levels of the study participants (N=390)

Level	Score	Frequency	Percentage
High	70 to 86	61	15.6%
Moderate	55 to 69	279	71.5%
Low	39 to 54	47	12%

Comparison between colleges

**Figure 1:**
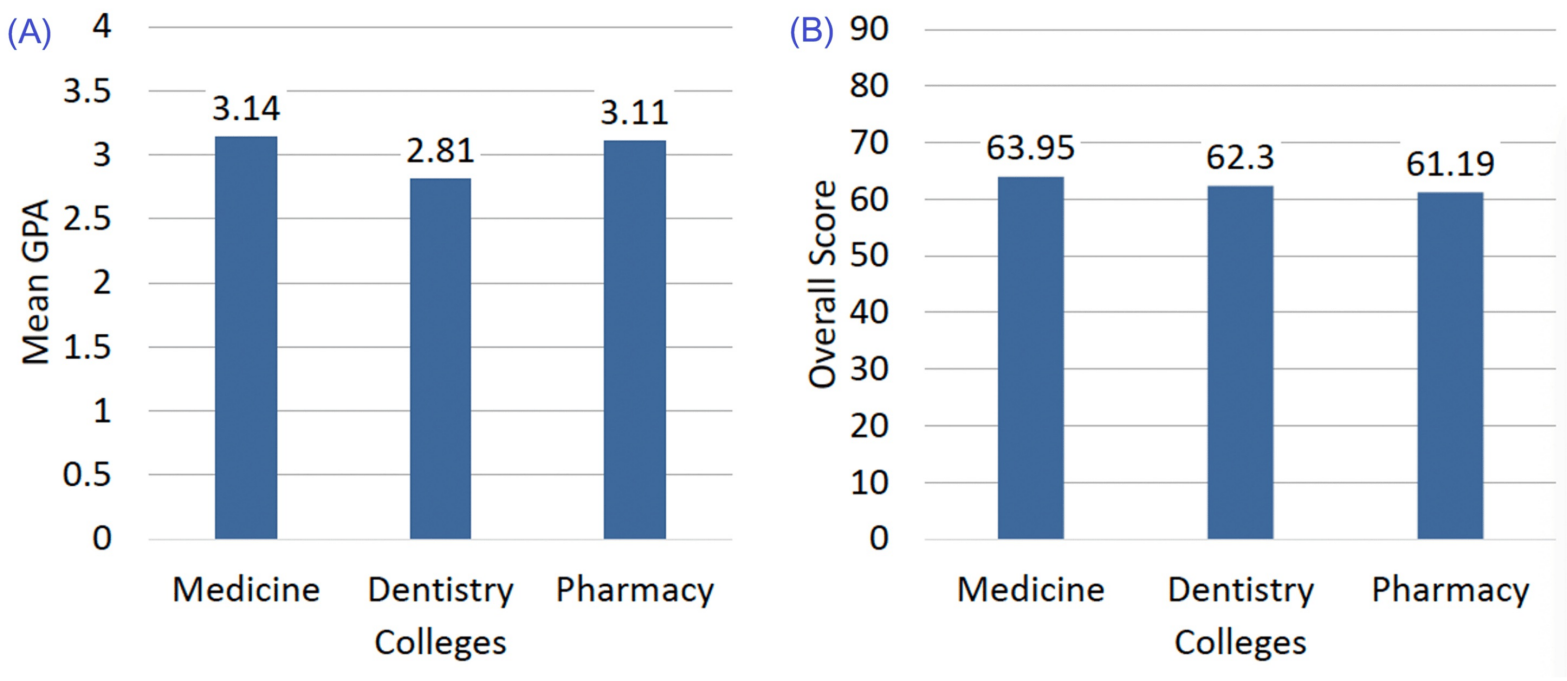
Comparison of overall mean GPA (A) and overall mean time-management (B) scores of the study population (N=390) (A) Overall mean GPA of students from Medicine, Dentistry, and Pharmacy colleges. (B) Overall mean time-management scores of students from Medicine, Dentistry, and Pharmacy colleges. GPA: Grade Point Average

A statistically significant difference in time-management scores was found between Medicine and Pharmacy students (p = 0.006), with medical students demonstrating slightly higher scores. No significant differences were observed between Dentistry and the other programs. Figure 1 shows the comparison of scores between colleges.

Correlation between time management and GPA

**Figure 2:**
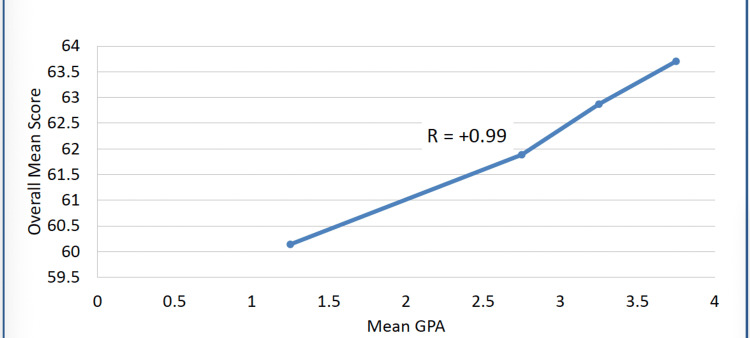
Relationship between time management scores and GPA GPA: Grade Point Average

As reflected in Figure 2, a correlation analysis demonstrated a very strong positive association between time-management scores and GPA (r = 0.99, p = 0.028). Because such a high correlation is uncommon in behavioral research, the analysis was independently repeated by two authors using both Pearson and Spearman methods, yielding identical results. Examination of the data distribution showed a markedly restricted GPA range, with the majority of students clustered within high academic categories, and limited variability in time-management scores. This restricted variance, combined with the homogeneity of the cohort, contributes to the magnitude of the observed correlation.

Demographic influences

No significant relationships were found between time-management scores and gender, high-school background, or academic level (preclinical vs clinical).

## Discussion

The findings indicate that most students demonstrated moderate time-management proficiency, consistent with prior studies from Turkey and the UAE [2-4], and with European research reporting predominantly moderate levels of time-management proficiency among university students [10,11]. This suggests that while students tend to employ some degree of structured time use, many may still lack the advanced planning strategies necessary to achieve peak academic performance.

A key finding in this study is the very strong positive correlation between time-management skills and GPA. Several mechanisms may help explain this relationship. Effective time management may reduce reliance on last-minute cramming, encourage distributed study practices, and allow students to allocate sufficient time for complex coursework. Additionally, planned study schedules may facilitate more consistent sleep patterns, reduce academic stress, and improve cognitive efficiency, all factors shown to support stronger academic performance. These mechanisms align with prior literature suggesting that time management functions not only as a behavioral skill, but also as a protective factor against stress, burnout, and academic overload [1,5,6].

The absence of significant differences between medical, dental, and pharmacy students is also noteworthy. Although these programs vary in content, they share similar academic intensity, clinical exposure, and continuous assessment structures. Such curricular similarities may place comparable time-management demands across programs, resulting in similar performance observed. This may explain why dentistry students, despite a unique clinical skill burden, did not differ significantly from their counterparts.

Interestingly, although gender-based differences in time-management skills have been reported internationally [1,5,12,13], such variation was not observed in the present study. These include standardized grading systems, similar expectations across programs, and cultural norms emphasizing academic diligence across genders. Such contextual features may contribute to a more uniform distribution of time-management behaviors.

Limitations

This study has several limitations that should be considered when interpreting the findings. The sample was predominantly female, which may influence the representativeness of the results. Data were collected from a single institution, limiting generalizability to other educational contexts. Additionally, reliance on self-reported GPA introduces potential reporting bias and limits the ability to infer causality from the observed associations. The academically homogenous nature of the cohort and the restricted GPA range may also have amplified the strength of the correlation observed. Future research should adopt multi-institutional and longitudinal designs, incorporate objective academic performance metrics, and explore qualitative perspectives to further elucidate the mechanisms and contextual factors underlying time-management behaviors.

## Conclusions

Time management emerged as a strong predictor of academic performance among medical, dental, and pharmacy students in this study. Given that most students demonstrated only moderate proficiency, institutions may benefit from integrating structured time-management training into their academic support systems, with targeted components focusing on short-range planning (daily scheduling and prioritization), long-range planning (semester mapping and deadline management), and time attitudes (addressing procrastination and enhancing motivation). While the strength of the association should be interpreted cautiously due to reliance on self-reported GPA and the single-institution design, improving students’ time-management capabilities remains a promising strategy for enhancing academic performance, reducing stress, and supporting long-term educational and professional success.
